# Physiological Roles of Carnosine in Myocardial Function and Health

**DOI:** 10.1093/advances/nmac059

**Published:** 2022-06-11

**Authors:** Jade V Creighton, Lívia de Souza Gonçalves, Guilherme G Artioli, Di Tan, Kirsty J Elliott-Sale, Mark D Turner, Craig L Doig, Craig Sale

**Affiliations:** Musculoskeletal Physiology Research Group, Sport, Health, and Performance Enhancement (SHAPE) Research Centre, School of Science and Technology, Nottingham Trent University, Clifton, Nottingham, United Kingdom; Department of Pediatrics, Nephrology, University of California, San Francisco, CA, USA; Department of Life Sciences, Manchester Metropolitan University, Manchester, United Kingdom; Natural Alternatives International, Inc., Carlsbad, CA, USA; Musculoskeletal Physiology Research Group, Sport, Health, and Performance Enhancement (SHAPE) Research Centre, School of Science and Technology, Nottingham Trent University, Clifton, Nottingham, United Kingdom; Department of Sport and Exercise Sciences, Institute of Sport, Manchester Metropolitan University, Manchester, United Kingdom; Centre for Diabetes, Chronic Diseases, and Ageing, School of Science and Technology, Nottingham Trent University, Clifton, Nottingham, United Kingdom; Centre for Diabetes, Chronic Diseases, and Ageing, School of Science and Technology, Nottingham Trent University, Clifton, Nottingham, United Kingdom; Musculoskeletal Physiology Research Group, Sport, Health, and Performance Enhancement (SHAPE) Research Centre, School of Science and Technology, Nottingham Trent University, Clifton, Nottingham, United Kingdom; Department of Sport and Exercise Sciences, Institute of Sport, Manchester Metropolitan University, Manchester, United Kingdom

**Keywords:** animals, β-alanine, carnosine, calcium transients, contractility, heart, humans, lipid peroxidation, metabolism, oxidative stress

## Abstract

Carnosine is a pleiotropic histidine-containing dipeptide synthesized from β-alanine and l-histidine, with the intact dipeptide and constituent amino acids being available from the diet. The therapeutic application of carnosine in myocardial tissue is promising, with carnosine playing a potentially beneficial role in both healthy and diseased myocardial models. This narrative review discusses the role of carnosine in myocardial function and health, including an overview of the metabolic pathway of carnosine in the myocardial tissue, the roles carnosine may play in the myocardium, and a critical analysis of the literature, focusing on the effect of exogenous carnosine and its precursors on myocardial function. By so doing, we aim to identify current gaps in the literature, thereby identifying considerations for future research.

## Teaser

**Statement of Significance:** Carnosine could perform important, yet different, roles within healthy and/or diseased hearts. Findings suggest that carnosine could improve calcium handling and, therefore, muscle contractility in a healthy model and oxidative stress, myocardial injury markers, and morphological and histological parameters in a diseased model. The translation of these findings into humans is, however, lacking, and considerable future work is required in this regard to determine whether carnosine has therapeutic potential.

## Introduction

Carnosine (β-alanine-l-histidine) is a pleiotropic histidine-containing dipeptide (HCD) abundant in skeletal muscle and the central nervous system—specifically, the olfactory bulbs [for a comprehensive review, see Boldyrev et al. (1)]. Carnosine was first isolated in the early 1900s (2) and has become well known for its role as an intracellular pH buffer, particularly in skeletal muscle, and subsequently, as an ergogenic aid to exercise capacity and performance (3, 4).

Various determinants of mammalian muscle carnosine content have been previously identified: diet, species, muscle fiber type, sex, age, training, and exercise (5). One of the key determinants of muscle carnosine content, particularly pertinent to this review, is the availability of carnosine and its constituent amino acids from the diet. The availability of dietary β-alanine is a key limitation to the in situ synthesis of carnosine (6), which can be largely obtained through the ingestion of meat/fish or via supplementation. In vegans, or in some vegetarians, however, the supply of β-alanine for carnosine synthesis is limited to its synthesis in the liver from uracil degradation or from supplemental sources if deemed acceptable. To highlight this, Everaert et al. (7) reported low skeletal muscle carnosine contents in vegetarian participants when compared with individuals consuming a fairly typical omnivorous Belgian diet. This makes sense given that the de novo synthesis of β-alanine in the liver would be augmented by the hydrolysis of dietary supplied HCDs from meat in omnivores but not in vegetarians, which would explain the significant differences in skeletal muscle carnosine content (8). The dietary availability of β-alanine might also be significantly influenced by cooking procedures, which can account for significant reductions in β-alanine availability.

Over the last 30 y, research has explored carnosine's therapeutic potential across various pathological conditions, including neurodegenerative diseases, diabetes, and in aging populations [for a review on this topic, see Artioli et al. (9)]. Emerging evidence has highlighted a promising effect of carnosine on healthy and diseased cardiovascular systems. This growing research suggests that the physiological roles of carnosine in myocardial tissue could involve the regulation of calcium handling and sensitivity, quenching of reactive oxygen species (ROS), detoxification of reactive aldehydes [including lipid peroxidation products and advanced-glycation end products (AGEs)], chelation of transition metal ions, and improvements in histological and hemodynamic parameters (1). This narrative review explores the influence of carnosine and its physiological and potentially therapeutic roles in the heart, critically examining data from studies concerning both healthy and diseased myocardial models. We have focused specifically on human models where literature is available, but data are limited in this regard as it is challenging to access human myocardial tissue. Therefore, literature involving animal myocardial tissue and other experimental models has been included. In cases where these data do not exist, we have extrapolated from other relevant tissues (i.e., skeletal muscle). This review also suggests some future directions for research in this area. Due to the heterogeneity of experimental designs (e.g., species, supplementation dosage, supplementation period, and experimental model), it was impossible to conduct a systematic review and meta-analysis.

## Myocardial Carnosine Concentration

Carnosine is found in high concentrations within skeletal muscle and the brain (**Table 1**). The intracellular concentration of carnosine within myocardial tissue has been suggested to be 0.1 mmol/kg wet weight, with the total concentration of HCDs being as high as 10 mM/kg wet weight (10). This is a plausible assumption in myocardial tissue since HCD concentrations are typically higher in fast-twitch glycolytic fibers and lower in slow-twitch oxidative fibers (11), meaning that we would not expect concentrations in the oxidative myocardial tissue to exceed those measured in the more glycolytic skeletal muscle, and concentrations of other amino acids in myocardial tissue fall within this low millimolar range (12, 13). Studies that have measured HCD concentrations within myocardial tissue are scarce, and there are discrepancies between those that have. Flancbaum et al. (14) identified the presence of carnosine in murine (mean ± SEM: 10.94 ± 3.12 μg/g), rat (mean ± SEM: 25.11 ± 3.22 μg/g), guinea pig (mean ± SEM: 17.39 ± 3.74 μg/g), and human (mean ± SEM: 10.12 ± 1.23 μg/g) myocardial tissue, whereas Jackson and Lenney (15) only confirmed the presence of carnosine within rat hearts and not human hearts using immunoreactivity. Chan et al. (16) and Liu et al. (17) measured endogenous carnosine in rat hearts to be 189.2 ± 3.5 μg/g (mean ± SEM) and 36.9 ± 13.61 μg/g (mean ± SEM). Interspecies variation and differences in the sensitivity and accuracy of the methodological techniques used may explain the inconsistencies between studies (18). In contrast to skeletal muscle, n-acetylcarnosine (an n-acetyl derivative of carnosine) may be the predominant HCD in mammalian myocardial tissue, with carnosine being present at a relatively low concentration (10, 19), although it is unclear why this may be the case. The additional n-acetyl group may allow n-acetylcarnosine to be more resistant to hydrolysis by carnosinase (20), or it may have a more fundamental role within myocardial tissue. The effect of supplementation on n-acetylcarnosine content has not been investigated; it would be interesting to identify whether its concentration can be increased via β-alanine or carnosine supplementation and, if so, whether this exerts any benefit to myocardial tissue. Therefore, a complete profile of the intracellular concentration of carnosine, and its associated derivatives, in myocardial tissue is warranted across a range of species, including humans.

**TABLE 1:** Carnosine content of skeletal muscle and the brain in various species

Study (reference)	Tissue	Species	Concentration
**Skeletal muscle**			
Harris et al. (21)	Vastus lateralis	Human	16.0 ± 7.2 mmol · kg^−1^ dry muscle (mean ± SD)
Flancbaum et al. (14)	Muscle	Rat	19.07 ± 6.77 μg/g (mean ± SEM)
		Mouse	8.03 ± 4.27 μg/g (mean ± SEM)
		Guinea pig	5.15 ± 0.41 μg/g (mean ± SEM)
Mannion et al. (22)	Quadriceps femoris	Human	20 ± 4.7 mmol · kg^–1^ dry muscle (mean ± SD)
Chan et al. (16)	Leg	Rat	874.1 ± 88.1μg/g (mean ± SEM)
**Brain**			
Margolis (23)	Olfactory bulb	Mouse	2.2 mM
	Whole brain (excluding olfactory bulb)	Mouse	Not detected
Flancbaum et al. (14)	Olfactory bulb	Rat	11.20 ± 5.04 μg/g (mean ± SEM)
		Mouse	31.10 ± 4.85 μg/g (mean ± SEM)
		Guinea pig	5.28 ± 2.49 μg/g (mean ± SEM)
	Hypothalamus	Rat	3.91 ± 0.71 μg/g (mean ± SEM)
		Mouse	25.97 ± 3.83 μg/g (mean ± SEM)
		Guinea pig	3.11 ± 0.95 μg/g (mean ± SEM)
	Pituitary	Rat	14.22 ± 3.41 μg/g (mean ± SEM)
		Guinea pig	1.56 ± 1.08 μg/g (mean ± SEM)
	Cerebrum	Mouse	7.60 ± 2.17 μg/g (mean ± SEM)

## Myocardial Carnosine Metabolism

**FIGURE 1:**
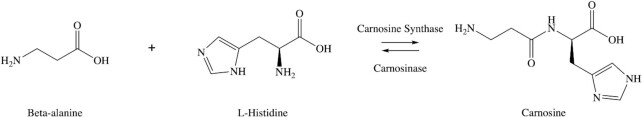
The chemical structure of β-alanine (C_3_H_7_NO_2_), l-histidine (C_6_H_9_N_3_O_2_), and carnosine (C_9_H_14_N_4_O_3_), and the metabolic pathway between carnosine and its constituent amino acids. (Created using Chem Draw (PerkinElmer).)

The major pathways of carnosine metabolism consist of hydrolysis to, and synthesis from, its constituent amino acids (**Figure 1**) and the activities of peptide transporters, β-alanine transporters, and transaminases.

### Carnosine synthase

The metabolic pathway of carnosine is regulated by carnosine synthase (CARNS1) and carnosinase activity (CN1 and CN2) (24). CARNS1, a member of the adenosine triphosphate (ATP)-grasp family, catalyzes β-alanine, l-histidine, and ATP into carnosine, adenosine diphosphate, and inorganic phosphate (25). Carnosine synthesis is dependent upon the availability of its constituent amino acids from the diet, either directly or from the hydrolysis of HCDs. The content of β-alanine in cells is substantially lower than l-histidine and the affinity for CARNS1 is also lower for β-alanine compared with l-histidine (*K*_m_ values of 1.0 to 2.3 mM and ∼17 μM) [rat brain (26); mouse olfactory pathway (27); rat central nervous system (28)], meaning that β-alanine availability is considered the rate-limiting precursor for carnosine synthesis in skeletal muscle cells (6). Although these data do not relate specifically to myocardial tissue, β-alanine may also be rate-limiting in the myocardium due to the abundance of l-histidine.

As mentioned previously, β-alanine is produced in the liver through the degradation of uracil and released into the bloodstream (29). The demand for β-alanine is assumed to far exceed this endogenous supply (1). Figure 2 in Artioli et al. (30) provides a detailed diagram of the β-alanine pathway and synthesis rate in the liver. Carnosine synthesis is dependent upon the availability of exogenous β-alanine ingested through the diet (meat extracts, poultry, and fish) (7, 8) or via supplementation. A typical Belgian omnivorous diet provides approximately 300 mg/d of β-alanine and a vegetarian/vegan diet will provide almost no β-alanine (31). Sufficient availability of β-alanine is considered essential for myocardial carnosine synthesis (32). Once synthesized, it is assumed that carnosine does not readily exit the cell in its intact form and can accumulate in the cell (33); nevertheless, carnosine content may wash out over time due to degradation.

**FIGURE 2:**
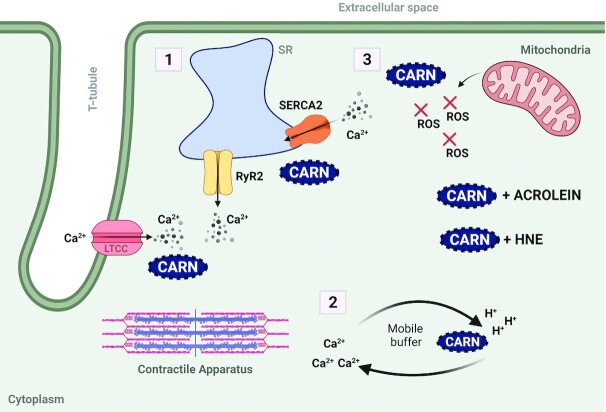
The main physiological roles of carnosine in myocardial function and health: an overview. (1) Carnosine (CARN) regulates EC coupling by influencing calcium (Ca^2+^) release from the SR via the RyR2 and Ca^2+^reuptake via SERCA2. (2) Carnosine acts as a mobile Ca^2+^/H^+^ buffer, transporting Ca^2+^ across the cytosol in an H^+^-coupled manner. (3) Carnosine prevents excessive accumulation of oxidative stress products (e.g., ROS) and acts as a scavenger to form covalent adducts with reactive aldehydes (e.g., acrolein and HNE) (created using BioRender.com). EC, excitation-contraction; HNE, 4-hydroxy-2-nonenal; LTCC, L-type calcium channel; ROS, reactive oxygen species; RyR2, ryanodine receptor; SERCA2, sarco(endo)plasmic reticulum Ca^2+^ ATPase; SR, sarcoplasmic reticulum.

### Carnosinases

Carnosine is degraded into its constituent amino acids by two isoforms of carnosinase: CN1 and CN2. CN1 is primarily expressed in human plasma with high specificity for carnosine and homocarnosine, whereas CN2, known initially as tissue carnosinase, has a broad substrate specificity and is ubiquitously expressed throughout central and peripheral human tissues (34–36). CN1 is present in animal and human myocardium (25, 37), although it is unclear whether this is simply a result of cross-contamination of circulating blood within myocardial tissue rather than it being directly expressed in the tissue (34, 35). The role of CN2 in the myocardium has not been confirmed given that there are inconsistencies in the literature regarding myocardial CN2 expression in humans. Lenney et al. (35) discovered CN2 activity in the human heart, whereas Teufel et al. (36) showed mRNA expression but no protein expression. Carnosinases optimally function at a pH of 7.5 to 8.5 (CN1) and 9.5 (CN2) (1, 24). Even though mRNA transcripts of CN2 are expressed in skeletal muscle (38, 39), the pH of skeletal [∼7.1 (40)] and cardiac [∼7.2 (41)] muscles are not optimal for CN2 activity. This suggests that, even if the enzyme is present in the tissue, it is not guaranteed to participate in the regulation of tissue HCD concentrations. This suggestion is consistent with the very low rates of carnosine degradation shown in human skeletal muscle washout studies. These results indicate that carnosine is a highly stable metabolite and carnosinases do not play a role within skeletal muscle myocytes (31).

### β-alanine transaminases

Alanine-glyoxylate aminotransferase 2 (also known as AGXT2 or β-alanine-pyruvate transaminase) and 4-aminobutyrate-2-oxoglutarate transaminase (also known as GABA-T or β-alanine-2-oxoglutarate transaminase) have been reported to be responsible for catalyzing the degradation of excess exogenous β-alanine into keto-acid malonate semi-aldehyde (MSA). MSA may be converted into acetyl-coenzyme A and subsequently enter the citric acid cycle (32), although this hypothesis requires further investigation. Oxidative muscles (e.g., myocardial tissue and soleus) have a higher expression of β-alanine transaminases when compared with more glycolytic muscles (e.g., gastrocnemius and tibialis anterior muscles). This may explain the lower concentrations of carnosine in myocardial tissue when compared with the high concentration found in skeletal muscle. The transaminase degradation process may limit the quantity of β-alanine available for carnosine synthesis. The importance of transaminases in myocardial β-alanine and carnosine homeostasis is unclear and requires further investigation, although the contribution of AGXT2 to overall carnosine content is likely to be small. Plasma and skeletal muscle β-alanine and carnosine concentrations were unaffected in AGXT2-knockout mice and in humans with a decreased AGXT2 activity genotype (42), and a nonsignificant elevation in circulating β-alanine was shown with Vigabatrin (Sabril; Lundbeck, Deerfield, IL, USA), a GABA-T inhibitor (32). This suggests that β-alanine homeostasis is either maintained by a tightly regulated system between GABA-T and AGXT2 (i.e., when one enzyme's activity is altered, the other enzyme can compensate) or AGXT2 activity in β-alanine and carnosine homeostasis is low and the system is dependent upon GABA-T activity. When both GABA-T and AGXT2 were inhibited with amino-oxyacetate, total HCD content in striated muscles (including myocardial and skeletal muscles) was increased 10-fold (32), suggesting that GABA-T, and not AGXT2, regulates endogenous β-alanine homeostasis. It would be interesting to investigate transaminase activity in response to exogenous β-alanine provision and whether they influence its availability and uptake into myocardium for a potential contribution to carnosine synthesis.

### Amino acid and nonspecific peptide transporters

Proton-coupled amino acid transporter (PAT1) and sodium and chloride-dependent taurine transporter (TauT) are likely to facilitate β-alanine transportation, and the proton-coupled oligopeptide transporter family (peptide transporter 1 (PEPT1), peptide transporter 2 (PEPT2), peptide-histidine transporter 1 (PHT1) and peptide-histidine transporter 2 (PHT2); collectively known as the solute carrier family 15) are responsible for nonspecific cellular transportation of peptides (43). The degree of their involvement in the cellular transportation of amino acids and peptides into the myocardium is unknown. β-alanine and taurine regulate the expression of the transporter TauT within skeletal muscle and decreased β-alanine uptake was shown in an immortalized mouse skeletal muscle cell line (C2C12) exposed to hypotaurine (a TauT inhibitor), suggesting that TauT may be the primary transporter of β-alanine in skeletal muscle (44). Due to the abundance of taurine in myocardial tissue (45), it can be postulated that β-alanine would also enter myocardial tissue via this transporter. Rodent studies have demonstrated a significant decrease in myocardial taurine content with β-alanine supplementation (46–51), suggesting competition between these two amino acids to use TauT for transportation into the myocardium, although this remains to be directly confirmed. The role of PAT1 in the myocardium may be primarily associated with cell growth regulation rather than amino acid transportation (52), suggesting that TauT may be the preferred transporter for the uptake of β-alanine into the myocardium.

## Physiological Roles of Carnosine in Myocardial Tissue

The proposed physiological roles of carnosine include intramuscular pH buffer, the regulation of intramuscular calcium handling and improved contractile apparatus sensitivity to calcium, improvements in histopathological and hemodynamic parameters, the quenching of ROS, metal ion chelation, and protection against lipid peroxidation and AGEs (1) (**Figure 2**). Much of the research into carnosine has focused on the implications in exercising human skeletal muscle [for a comprehensive review on this topic, see Matthews et al. (40)]. The first study looking at the physiological effect of carnosine on cardiovascular function demonstrated a decrease in systemic pressure in canines injected with carnosine (53). It has been suggested that carnosine plays different roles within different tissues, the same role in different tissues, and even multiple roles in the same tissue depending upon the health of that tissue. This section of the review will focus on the mechanistic action of endogenous and exogenous carnosine in both healthy and diseased myocardial models.

### Intramuscular pH buffer and energy metabolism

Carnosine is recognized as an intracellular pH buffer due to the imidazole ring on the histidine residue of carnosine having a pKa of 6.83 (54). Although carnosine's buffering role is highly relevant to skeletal muscle, its role in myocardial tissue remains unclear. The absence of hydrogen ion accumulation, alongside the dependence on mitochondrial oxidative metabolism to maintain homeostasis (55), suggests that any role of carnosine within healthy myocardial tissue may not primarily relate to intracellular pH buffering and energy metabolism. In healthy *CARNS1*-knockout (*CARNS1^–^/^–^*) rats, the absence of endogenous HCDs (such as carnosine) did not impair mitochondrial function or oxygen consumption and hydrogen peroxide release was not different between the *CARNS1^–^/^–^* rats and wild-type controls (56). This may be attributed to the fact that oxidative stress, which can lead to mitochondrial dysfunction, was not increased in the absence of carnosine. Although carnosine may not exert these effects in healthy myocardial tissue, it could have a protective role under diseased conditions. When *ATPGD1-*transgenic mice overexpressing *CARNS1* were exposed to myocardial ischemia, an increase in endogenous carnosine attenuated ischemia/reperfusion-induced changes in intracellular pH (57). In isolated rat hearts exposed to cardioplegia (a pharmacological solution used to cease cardiac activity temporarily), an HCD supplement (consisting of carnosine, l-histidine, and acetylcarnosine) resulted in reduced lactate content and increased oxygen consumption (58). This suggests that the hearts may have experienced increased oxidative energy metabolism and relied less on glycolysis when supplemented with HCDs.

### Calcium handling and contractility

Carnosine has been described as an inotropic agent that can potentiate myocardial contractility (59). Excitation-contraction (EC) coupling and systolic (contraction) and diastolic (relaxation) functions are regulated by the dynamics of cytoplasmic concentrations of calcium ions within cardiomyocytes (60–62). Myocardial contractility depends upon the availability of calcium and the sensitivity of myofilaments to calcium (62).

Carnosine has been suggested to influence intracellular calcium concentrations and myocardial contractility. Gonçalves et al. (56) recently developed a novel *CARNS1^–^/^–^* rat strain to investigate the absence of HCDs (including carnosine) on myocardial function. In isolated cardiomyocytes, the lack of carnosine and HCDs reduced sarcomere's maximal shortening, re-lengthening, and velocity measurements, affecting the overall contractility of the cardiomyocytes. These results, alongside impairments shown in *in vivo* functional data [i.e., electrocardiogram (ECG) and echocardiogram], indicate that the absence of carnosine in these animals could result in impairments to calcium handling. In addition, decreased calcium amplitude was shown, although there was no change in calcium release or sarco(endo)plasmic reticulum Ca^2+^ ATPase (SERCA2) expression. The study showed increased isovolumetric contraction time and reduced left ventricular ejection fraction and shortening fraction in *CARNS1^–^/^–^* rats, indicating an impairment in systolic function, whereby the left ventricle has difficulty pumping blood out of the heart, increasing the volume of blood remaining in the ventricle at the end of the contraction. Despite this, cardiac output was unaffected, suggesting that the heart might be able to compensate. *CARNS1^–^/^–^* rats display a reduced e′ wave and an increased E:e′ ratio, suggesting left ventricular relaxation impairments and a diminished diastolic function. This indicates that endogenous HCDs (since we cannot be sure from rodent studies that the effect is limited to carnosine or whether this relates to anserine or their acetylated analogues) are essential for myocardial calcium handling and contractility under healthy conditions (i.e., with no cardiometabolic disease), given that the myocardium was unable to function as well in these rats when compared with wild-type controls.

Exogenous carnosine has been shown to benefit calcium handling and myofilament sensitivity regulation (63) in a dose-dependent manner in healthy rat hearts and ventricular pig tissue (59). This regulation occurs at two cellular sites of action: the sarcoplasmic reticulum and the ryanodine receptors (RyR) (64). RyR2 (the myocardium isoform) is a significant ion channel responsible for calcium release from the sarcoplasmic reticulum to the sarcoplasm during myocardial contraction (65). Carnosine may influence the opening of the RyR2; procaine, an anesthetic that blocks calcium release from RyR2, blocks the tension response to 40 mM carnosine (59). The addition of two physiological concentrations of carnosine (8 and 16 mM) improved RyR1 (the skeletal muscle isoform) calcium sensitivity and increased calcium release in mechanically skinned human skeletal muscle, directly enhancing contractile apparatus force production (66). Even though this was in skeletal muscle, we may see similarities in myocardial tissue as the myocardial muscle is also striated, contains calcium, and is involved in muscle contractility. In chemically skinned cardiomyocytes, carnosine increased calcium release from the sarcoplasmic reticulum, but did not affect the rate of calcium reuptake (64), and enhanced myofibrillar calcium sensitivity (63). Chemically skinned tissue lacks a functional sarcoplasmic reticulum. Still, when the chemically skinned skeletal muscle was compared with mechanically skinned skeletal muscle, the effects on contractile apparatus properties was not detectably different (66). Overall, in healthy models, carnosine may induce calcium release from the sarcoplasmic reticulum, increasing intracellular calcium availability and contractile apparatus sensitivity for calcium, subsequently affecting myocardial contractions. Supplementation with carnosine or its intracellular synthesis may be essential for these effects in a myocardial model; no alterations in myocardial contractility were noted when isolated rat hearts were independently treated with β-alanine or l-histidine (59) and no functional changes in ECG parameters were shown when healthy rats were supplemented with β-alanine only for 6 wk (67).

Altered calcium homeostasis is a primary contributor to myocardium pathophysiology, resulting in contractile dysfunction (20, 68). Therefore, carnosine's role in calcium handling and responsiveness has potential therapeutic applicability in myocardial tissue (62). Two weeks of carnosine supplementation (10 mg · kg^–1^ · d^–1^) attenuated detrimental ECG changes in rats exposed to adriamycin (69, 70), although 15 mM of carnosine did not improve contractility in isolated rat hearts exposed to hypoxia and reoxygenation (71). Interestingly, an impairment in intracellular calcium homeostasis has been shown in doxorubicin (DOX)-treated rats supplemented with β-alanine for 3 to 4 weeks (48). The β-alanine–treated rats at 48 h post-DOX displayed an increase in calcium concentrations that exceeded the increase seen in the nonsupplemented controls. This study also showed a decrease in calcium uptake into the sarcolemmal vesicles, enhancement in the inhibition of sarcolemmal calcium uptake and inhibition in ATP-dependent calcium uptake in isolated vesicles. Although interesting, these results should be interpreted with caution, since sarcoplasmic reticulum membranes and the lack of oxalate-facilitated calcium transportation in the myocardial preparations do not represent a physiological myocardial model.

The role of β-alanine supplementation may not be related to myocardial contractility in a diseased model. Allo et al. (47) showed, using rat hearts exposed to an ischemic occlusion and reperfusion model, that a decrease in infarct size–risk area ratio (calculated by dividing infarct zone volume by risk zone volume) with β-alanine supplementation did not correlate with changes in contractile function. The authors attributed this to the reduction in myocardial taurine concentration rather than a direct effect of β-alanine on the myocardium, although they did not measure or consider the potential roles of carnosine and n-acetylcarnosine. Therefore, exogenous carnosine and β-alanine may not influence calcium handling or myocardial contractility in a diseased myocardial model.

The ability of carnosine to buffer intramuscular pH may indirectly improve calcium handling. Carnosine can regulate calcium gradients by acting as a diffusible cytoplasmic calcium/hydrogen ion exchanger in isolated rat ventricular cardiomyocytes. This “pump” creates functional calcium gradients in response to local pH changes in the cardiomyocytes and transports calcium across the cytosol in a hydrogen ion–coupled manner. Calcium is released from the sarcoplasmic reticulum and dissipated across the cell to the sarcomeres, and hydrogen ions are released from the sarcomeres and dissipated across the cell (72). Many cellular processes activated by calcium, such as EC coupling, are inhibited by hydrogen ions. Calcium and hydrogen ions compete at the troponin binding site, limiting the ability of the contractility machinery to operate effectively (73). It should be noted that this experiment was conducted at room temperature rather than physiological temperature, which may have affected the behavior of the contractile apparatus. Nevertheless, cytoplasmic calcium/hydrogen ion-coupling can help sustain calcium activation during a metabolic challenge. Therefore, an increase in carnosine availability may increase calcium delivery to, and hydrogen removal from, the sarcomeres, potentially improving contractile function (72).

### Oxidative stress

Carnosine has been characterized as an antioxidant and antiglycator (74, 75) as it can neutralize and reduce oxidative reactivity. Many components of the myocardium are redox (oxidation-reduction reaction) sensitive; ROS are required to maintain homeostatic regulatory functions in cardiomyocytes, including cardiomyocyte development and maturation, calcium handling, EC coupling, and vascular tone (76, 77). Mitochondria and the sarcoplasmic reticulum produce ROS as by-products of myocardial oxidative metabolism (78, 79). Uncontrolled and excessive accumulation of oxidative stress, due to endogenous and exogenous stress, leads to an imbalance between these products and the endogenous antioxidant defense mechanisms, subsequently causing oxidative damage to lipids, proteins, and DNA (79–82). The low molecular weight (226 g/mol) and water solubility of carnosine make this dipeptide a “model” antioxidant (77). Dupin et al. (83) provided the first evidence of the antioxidant properties of carnosine: in skeletal muscle, 25 mM of carnosine inhibited the accumulation of lipid peroxidation end products in the sarcoplasmic reticulum and decreased malonic dialdehyde concentrations. Additionally, carnosine can form complexes with first-transition metals (e.g., Cu^2+^, Zn^2+^, and Fe^2+^), preventing the metal ions from contributing to the production of free radicals through the Fenton reaction, and thus reducing oxidative stress (77, 84). Intracellular AGEs further increase oxidative stress by reacting with transition metal ions (84). By buffering these metal ions, carnosine may also act as an AGE inhibitor, preventing AGE formation and glycoxidation (36, 84). Indeed, carnosine has recently been shown to prevent 65–90% of AGE and advanced lipid end product (ALE) protein adduct formation in skeletal muscle cells under metabolic stress (85), suggesting the potential for a similar role in cardiomyocytes.

Carnosine is considered a more effective scavenger of singlet oxygen than histidine, suggesting the intact dipeptide is required to improve the functional recovery of ischemic myocardium (86). The intact dipeptide is also necessary to achieve the most effective scavenging response of lipid peroxidation products [e.g., 4-hydroxy-2-nonenal (HNE) and trans-2-hexenal] (87). This suggests that radical scavenging ability can mainly be attributed to the N-terminus on the l-histidine residue (the imidazole ring); non-histidine-containing amino acids had limited interaction with the aldehydes (87). l-Histidine supplementation produces a greater scavenger response when compared with β-alanine, and β-alanine is relatively ineffective. Carnosine supplementation produced the most reactive response, suggesting that the β-alanine amino group is also needed to maximize aldehyde scavenging (87, 88); the constituent amino acids must work synergistically to achieve this. The lack of B-alanyl residue in n-acetylcarnosine may explain its low reactive scavenging ability compared with carnosine (88).

Carnosine can form covalent adducts with reactive aldehydes. The presence of aldehyde conjugates has been confirmed in humans. Baba et al. (89) and Bispo et al. (90) showed that carnosine can form stable and structurally distinct conjugates with HNE, 4-hydroxy-2-hexenal (HHE), and acrolein, and these aldehyde conjugates are subsequently excreted in the urine. An increase in carnosine-acrolein adduct concentrations in exercising skeletal muscle was shown in humans supplemented with β-alanine. This indicates that skeletal muscle is a site for adduct formation and increasing carnosine content can enhance reactive-aldehyde scavenging by carnosine (91). The ability of carnosine to block the formation of catechoaldehyde protein adducts has been demonstrated in myocardial tissue taken from patients undergoing elective heart surgery. Pretreatment of mitochondria with 1 mM carnosine showed a concentration-dependent reduction in catechol-modified adducts and attenuated the decrease in state 3 respiration seen with 3,4-dihydroxyphenylacetaldehyde (DOPAL) (92). Similar results have been shown in animal models. In *ATPGD1-*transgenic mice overexpressing *CARNS1*, an increase in endogenous carnosine protected the heart from damage caused by aldehydes (57), and in isolated adult mouse cardiomyocytes, the pretreatment of 1 mM carnosine protected the myocytes from aldehyde-induced hypercontracture (93).

Under healthy conditions, endogenous carnosine is unlikely to fulfil an antioxidant role in cardiomyocytes. Oxidative stress was not increased when myocardial tissue was depleted of endogenous carnosine and HCDs in a cardiometabolically healthy rat model; there was no change in hydrogen peroxide release or protein carbonyl concentrations in the myocardial tissue of *CARNS1^–^^/^^–^* rats (56). The physiological endogenous antioxidant defense mechanism is sufficient to maintain homeostatic redox; additional exogenous antioxidants, such as carnosine, provide no capacity for improvement. If carnosine quenched oxidative stress mediators in a healthy environment (where appropriate levels of oxidation products are required for normal function), the homeostatic balance might move towards an excess of antioxidants, and myocardial function and regulation may be disrupted. In a diseased myocardial model, an excess of ROS decreases the capability of the antioxidant system—an effective cellular antioxidant response can no longer be produced and additional antioxidant resources are required to improve defense mechanisms (78).

A summary of the literature investigating the effect of exogenous carnosine and β-alanine on oxidative stress markers in healthy and diseased myocardial models is presented in **Table 2**. There is still a significant gap in the literature for clinical studies in human participants supplemented with β-alanine or carnosine. The bulk of the support for carnosine as an antioxidant has been conducted in *in vitro* and experimental animal models (94), making the translation of beneficial findings into humans challenging (76). If carnosine can ameliorate myocardial oxidative stress, we need to establish the direct effect this has on myocardial function and address why it is necessary to improve oxidative stress in pathological conditions. The potential link between ROS and myocardial calcium transients and contractility is of great interest to future research and raises several critical questions regarding carnosine's role. Anti-ischemic activity may be influenced by the effects of carnosine on ROS concentrations (95). An increase in ROS may disrupt calcium handling and myofilament sensitivity to calcium, impair myocardial contractile function, and have detrimental effects on overall myocardial metabolism (96, 97). These findings pose several questions, including: Does this link explain why improving oxidative stress is vital to help maintain the regulatory functions of a diseased cardiovascular system? Furthermore, a correlation has been shown between singlet oxygen quenching ability*in vitro* and myocardial functional recovery with carnosine; can the improvements in hemodynamic parameters be explained by the scavenging of ROS (86)? A large-scale study that measures all associated mechanisms of carnosine in the myocardium in both healthy and diseased models is warranted to further look at these questions and establish the exact roles carnosine plays in these myocardial models.

**TABLE 2:** Summary of studies assessing the effect of exogenous carnosine, and its constituent amino acids, on oxidative stress parameters^1^

			Supplementation protocol		
Study (reference)	Species	Experimental model	Dosages(s)	Duration	Main results
Harada et al. (48)	Rat	Doxorubicin (5 mg/kg for 1 or 48 h)	3% β-alanine	3–4 wk	β-alanine supplementation increased tissue MDA in doxorubicin-treated rats but did not change tissue GSSG
Lee et al. (86)	Rat	40-min ischemia + 30-min reperfusion	1 mM carnosine, 1 mM l-histidine, or 10 mM l-histidine	20 min	One millimolar of carnosine was more effective at scavenging singlet oxygen compared with 1 mM or 10 mM of l-histidine
Parildar et al. (98)	Rat	Aging	3% β-alanine	6 wk	MDA and DC concentrations and AA- and NADPH-induced lipid peroxidation were increased in the hearts of aged rats but there were no changes in GSH, vitamin E, or vitamin C concentrations, or in SOD, GSH-Px, or GST activities. Cardiac MDA and DC concentrations and the antioxidant system did not further change in the hearts of β-alanine–treated aged rats. AA- and NADPH-induced lipid peroxidation increased in the heart of aged rats treated with β-alanine
Aydın et al. (99)	Rat	Ageing (young vs. old)	250 mg · kg^–1^ · d^–1^ carnosine	1 mo	Aged rats experienced an increase in MDA and DC concentrations but no differences in enzymatic and nonenzymatic antioxidant elements when compared to the young rats. Carnosine supplementation had no effect on cardiac oxidative stress parameters in young or aged rats
Dursun et al. (69)	Rat	Adriamycin (single dose of 16 mg/kg on day 14)	10 mg · kg^–1^ · d^–1^ carnosine	2 wk	Carnosine supplementation increased plasma CAT activity in the rats not treated with adriamycin. Adriamycin decreased plasma SOD, GSH-Px, and CAT activities; the addition of carnosine supplementation was able to maintain these activities at normal levels. Carnosine supplementation prevented the increase in plasma MDA that was seen with adriamycin
Özdoğan et al. (70)	Rat	Adriamycin (4 doses over 8 d)	10 mg · kg^–1^ · d^–1^ carnosine	2 wk	Carnosine supplementation prevented the increase in plasma lipid peroxidation that was seen with adriamycin. Plasma SOD, GSH-Px, and CAT activity were decreased with adriamycin. Carnosine supplementation was able to maintain normal concentrations of these antioxidants when added to the adriamycin group
Pansani et al. (51)	Rat	Healthy	3% β-alanine	30 d	β-alanine supplementation showed a higher concentration of tissue LH and lower activity of tissue CAT and GSH-Px
Kalaz et al. (100)	Rat	Healthy or stress protocol (immobilization and 4°C cold room for 1 h/d for 5, 7, or 21 d)	250 mg · kg^–1^ · d^–1^ carnosine	30 min prior to stress protocol	Cardiac concentrations of MDA, PC, DC, and NT and nonenzymatic and enzymatic antioxidants were not affected by the stress protocol. The addition of carnosine in the stress group did not have any effect on these markers—carnosine only caused a decrease in GSH-Px. Carnosine supplementation had no effect on these markers in physiologically healthy hearts
Evran et al. (101)	Rat	Isoproterenol	250 mg · kg^–1^ · d^–1^ carnosine	2 or 12 d	Carnosine pretreatment had no effect on plasma MDA and PC concentration but did increase FRAP values. Twelve days of carnosine pretreatment decreased cardiac MDA, DC, and PC concentrations and increased GSH concentrations and the activities of SOD and GSH-Px
Kumral et al. (102)	Rat	Doxorubicin (single dose of 30 mg/kg on day 8)	250 mg · kg^–1^ · d^–1^ carnosine	12 d	Carnosine supplementation decreased doxorubicin-induced oxidative stress (TBARS, PC, and DC concentrations) in cardiac tissue. GSH decreased with doxorubicin but this was increased with the addition of carnosine supplementation. GSH-Px activity remained unchanged
Hou et al. (103)	Rat	30-min coronary artery occlusion	100 mM β-alanine	30 d	The increase in cardiac MDA and intracellular ROS induced by the occlusion model was decreased with the addition of β-alanine supplementation. The decrease in cardiac SOD and CAT activity, and GSH and GSH-Px concentrations induced by the occlusion model was increased with the addition of β-alanine supplementation

1 AA, ascorbic acid; CAT, catalase; DC, diene conjugate; FRAP, ferric reducing ability of plasma; GSH, (reduced) glutathione; GSH-Px, glutathione peroxidase; GSSG, oxidized glutathione; GST, glutathione transferase; LH, lipid hydroperoxide; MDA, malondialdehyde; NT, nitrotyrosine; PC, protein carbonyl; ROS, reactive oxygen species; SOD, superoxide dismutase; TBARS, thiobarbituric acid reactive substances.

### Hemodynamic parameters

Endogenous HCDs may directly impact, or be influenced by, cardiovascular risk factors such as hypertension. Lower levels of HCDs (a 35% decrease) were shown in the left ventricular myocardial tissue of hypertensive rats compared with normotensive rats (19). This may explain why supplementation has a potential beneficial effect on hemodynamic parameters in diseased myocardial models (i.e., when there is the capacity to increase HCD content) and no impact upon these parameters when the myocardial tissue is healthy and contains an adequate concentration of HCDs. Johnson and Hammer (19) found no detectable levels of carnosine in either rat model, suggesting that carnosine may not be the predominant HCD in myocardial tissue.

There are contradictory findings regarding the vasodilatory properties of exogenous carnosine. The dose-dependent, vasodilatory effect of exogenous carnosine was demonstrated on isolated rat aortic rings (104). Carnosine provoked significant, sustained contractures in rabbit saphenous vein rings. This effect was specific to carnosine, as its constituent amino acids were ineffective, and is considered to be attributed to the zinc/carnosine complex (105, 106). Other mechanisms of the antihypertensive action of carnosine may be mediated via the histamine H1 receptors (107) and the carnosine-histamine-histidine pathway (108). A summary of the research investigating the effect of exogenous carnosine, and its constituent amino acids, on hemodynamic parameters in animal models is displayed in **Table 3**. There is very little knowledge concerning the influence of carnosine on hemodynamic parameters in humans. Midoh and Noguchi (109) showed that both a single intake and a 2-wk daily intake of chicken soup increased peripheral blood flow. No changes were noted in heart rate, systolic blood pressure, or diastolic blood pressure, and the authors did not suggest other mechanisms that may have induced this increase. Although this study was not directly focused on carnosine, we know that meat extracts are rich in amino acids and peptides, including carnosine. Therefore, these results may indicate a potential role of carnosine in hemodynamic regulation in humans.

**TABLE 3:** Summary of studies assessing the effect of exogenous carnosine, and its constituent amino acids, on hemodynamic parameters^1^

			Supplementation protocol		
Study (reference)	Species	Experimental model	Dosages(s)	Duration	Main results
Allo et al. (47)	Rat	45-min coronary artery occlusion + 120-min reperfusion	3% β-alanine	4–28 d	β-alanine supplementation did not affect hemodynamic parameters in a diseased model
Lee et al. (86)	Rat	40-min ischemia + 30-min reperfusion	1 mM carnosine, 1 mM l-histidine, or 10 mM l-histidine	20 min	The LVDP recovery of 1 mM carnosine-treated ischemic hearts improved more than untreated and 10 mM histidine-treated ischemic hearts; 1 mM carnosine and 10 mM histidine improved dP/dt recovery but did not improve coronary flow or HR; 1 mM histidine improved HR recovery but did not improve dP/dt, coronary flow, or LVDP recovery
Ririe et al. (104)	Rat	Healthy	0.625 – 20 mM carnosine, l-histidine, or β-alanine	30 min	Carnosine increased vasodilation in a dose-dependent manner. l-histidine and β-alanine had no vasodilatory effect. β-alanine increased vascular smooth muscle tone in a dose-dependent manner
Niijima et al. (110)	Rat	Normotensive or hypertensive (2×/wk DOCA + NaCl)	0.1 mg or 1 mg carnosine	5 wk	Carnosine had no effect on systolic blood pressure in normotensive rats (116 mmHg vs. 112 mmHg at 0 and 5 wk). Systolic blood pressure increased in the untreated hypertensive rats (114 mmHg vs. 198 mmHg at 0 and 5 wk). Carnosine supplementation decreased the rise in systolic pressure seen with hypertension (0.1 mg carnosine: 115 mmHg vs. 163 mmHg at 0 and 5 wk; 1 mg carnosine: 113 mmHg vs. 148 mmHg at 0 and 5 wk)
Zieba et al. (111)	Rabbit	Doxorubicin (2 mg · kg^–1^ · wk^–1^ for 7 wk)	100 mg · kg^–1^ · d^–1^ carnosine	9 wk	MAP, CI, and SI decreased with doxorubicin but the addition of carnosine with doxorubicin increased the levels to similar values seen in the untreated and treated groups not exposed to doxorubicin. There was no change in HR or TPR in any of the groups. Carnosine had no effect on haemodyanmic parameters in healthy rabbits not exposed to doxorubicin
Abebe and Mozaffari (46)	Rat	Endothelial-intact or endothelial-denuded	3% β-alanine	3 wk	β-alanine supplementation impaired the relaxation responses of blood vessels to adenosine agonists
Dursun et al. (69)	Rat	Adriamycin (single dose of 16 mg/kg on day 14)	10 mg · kg^–1^. d^–1^ carnosine	2 wk	The MAP and LVDP decrease seen with adriamycin was maintained by the addition of carnosine. Similar results were seen with dP/dt; however, this was not significant
Özdoğan et al. (70)	Rat	Adriamycin (4 doses over 8 d)	10 mg · kg^–1^ · d^–1^ carnosine	2 wk	The decrease in LVDP and ±dP/dt induced by adriamycin was increased with the addition of carnosine
Pansani et al. (51)	Rat	Healthy	3% β-alanine	30 d	β-alanine supplementation decreased LVSD, HR, EF, and %FS and increased E/A ratios compared with the untreated group
Stefani et al. (112)	Rat	Coronary heart failure induced by myocardial infraction surgery	250 mg · kg^–1^ · d^–1^ β-alanine + 55–75 mg · kg^–1^ · d^–1^ l-histidine	8 wk	No change in hemodynamic parameters with supplementation

1 CI, cardiac index; dP/dt, first derivative of left ventricular pressure; E/A, relationship between the E and A waves; EF, ejection fraction; FS, fractional shortening; HR, heart rate; LVDP, left ventricular developed pressure; MAP, mean arterial pressure; SI, stroke index; TPR, total peripheral resistance.

### Myocardial injury markers, morphological and histological parameters

A summary of the literature investigating the effect of carnosine and β-alanine supplementation on myocardial injury markers and morphological and histological characteristics are displayed in **Tables 4** and **5**. Under healthy conditions, β-alanine and carnosine supplementation do not affect these parameters in the heart. The lack of myocardial injury may explain this; parameters are within their physiological range, and, therefore, there is no capacity or need for improvement.

**TABLE 4:** Summary of studies assessing the effect of exogenous carnosine and β-alanine on myocardial injury markers^1^

			Supplementation protocol		
Study (reference)	Species	Experimental model	Dosages(s)	Duration	Main results
Alabovsky et al. (113)	Rat	40-min ischemia + 12-min reperfusion	2 mM carnosine, 5 mM carnosine, 10 mM carnosine, or 10 mM acetyl-carnosine	15 s prior to ischemia and during the 40-min ischemic period	Carnosine and acetyl-carnosine supplementation reduced the level of myoglobin released from the heart during ischemia/reperfusion; 10 mM carnosine supplementation decreased the release of myoglobin and nucleosides during ischemia/reperfusion but 2 mM carnosine had no effect
Dursun et al. (69)	Rat	Adriamycin (single dose of 16 mg/kg on day 14)	10 mg · kg^–1^ · d^–1^ carnosine	2 wk	Carnosine supplementation reduced the increase in plasma CK induced by adriamycin
Özdoğan et al. (70)	Rat	Adriamycin (4 doses over 8 d)	10 mg · kg^–1^ · d^–1^ carnosine	2 wk	Carnosine supplementation reduced the increase in plasma markers (CK, AST, ALT, and LDH) induced by adriamycin
Pansani et al. (51)	Rat	Healthy	3% β-alanine	30 d	β-alanine supplementation had no effect on MMP-2 and MMP-9 values
Evran et al. (101)	Rat	Isoproterenol	250 mg · kg^–1^ · d^–1^ carnosine	2 or 12 d	Twelve days of carnosine supplementation did not affect plasma cTnT or CK activities but did decrease plasma LDH and AST activities. Two days of carnosine supplementation did not affect these plasma markers
Al-Rasheed et al. (114)	Rat	TiO_2_ (600 mg/kg or 1 g/kg)	200 mg · kg^–1^ · d^–1^ carnosine	3 wk	Carnosine supplementation decreased blood measurements (myoglobin, troponin, CK-mB, and CRP) and cardiac capase 3 when compared with control and TiO_2_-only groups
Kumral et al. (102)	Rat	Doxorubicin (single dose of 30 mg/kg on day 8)	250 mg · kg^–1^ · d^–1^ carnosine	12 d	Carnosine supplementation reduced the increase in serum cTn1 induced by doxorubicin
Keskin et al. (115)	Rat	30-min ischemia + 60-min reperfusion of the infrarenal abdominal aorta	250 mg · kg^–1^ · d^–1^ carnosine	10 min prior to the end of ischemia	Carnosine supplementation improved AST and LDH levels in the ischemia/reperfusion model

1 ALT, alanine aminotransferase; AST, aspartate aminotransferase; CK, creatine kinase; CK-mB, creatine kinase isoenzyme; CRP, C-reactive protein; cTn1, cardiac troponin 1; cTnT, cardiac troponin T; LDH, lactate dehydrogenase; MMP, matrix metalloproteinases; TiO_2_, titanium dioxide.

**TABLE 5:** Summary of studies assessing the effect of exogenous carnosine, and its constituent amino acids, on myocardial histological and morphological parameters^1^

			Supplementation protocol		
Study (reference)	Species	Experimental model	Dosages(s)	Duration	Main results
Allo et al. (47)	Rat	45-min coronary artery occlusion + 120-min reperfusion	3% β-alanine	4–28 d	β-alanine supplementation reduced the infarct size area ratio by 57%
Zieba et al. (111)	Rabbit	Doxorubicin (2 mg · kg^–1^ · wk^–1^ for 7 wk)	100 mg · kg^–1^ · d^–1^ carnosine	9 wk	A smaller degree of damage was noted in the doxorubicin hearts treated with carnosine when compared with doxorubicin hearts not treated with carnosine. No differences were shown between control hearts (no doxorubicin) and control hearts treated with carnosine
Dursun et al. (69)	Rat	Adriamycin (single dose of 16 mg/kg on day 14)	10 mg · kg^–1^ · d^–1^ carnosine	2 wk	Carnosine supplementation improved the edema, organizational disturbance, and myocardial injury abnormalities induced by adriamycin
Pansani et al. (51)	Rat	Healthy	3% β-alanine	30 d	β-alanine supplementation decreased LVWT, LVWT/LVDD, and myocardial cross-sectional area
Kumral et al. (102)	Rat	Doxorubicin (single dose of 30 mg/kg on day 8)	250 mg · kg^–1^ · d^–1^ carnosine	12 d	Carnosine supplementation decreased the histological damage seen with doxorubicin (mild degree of interstitial edema and lymphocyte infiltration, irregular clusters of myocardial fibers, and necrotic changes)
Keskin et al. (115)	Rat	30-min ischemia + 60-min reperfusion of the infrarenal abdominal aorta	250 mg · kg^–1^ · d^–1^ carnosine	10 min prior to the end of ischemia	Carnosine supplementation had no effect on the histological parameters
Hou et al. (103)	Rat	30-min coronary artery occlusion	100 mM β-alanine	30 d	β-alanine supplementation reduced the increase in infarct size induced by ischemia/reperfusion. Apoptosis increased with ischemia/reperfusion but was decreased with β-alanine (not fully recovered)
Stefani et al. (112)	Rat	Coronary heart failure induced by myocardial infraction surgery	250 mg · kg^–1^ · d^–1^ β-alanine + 55–75 mg · kg^–1^ · d^–1^ l-histidine	8 wk	β-alanine supplementation did not affect structural and morphological parameters or infarct size in the diseased model

1 LVDD, left ventricular end-diastolic diameter; LVWT, left ventricular posterior wall thickness.

### Methodological limitations

The cardioprotective effects of carnosine have primarily been researched within animal models; there is little evidence in human myocardial tissue. It is therefore difficult to extrapolate these findings to the human population. First, CN1, the enzyme responsible for the hydrolysis of carnosine into β-alanine and l-histidine, is absent in non-primate mammals (34). The results in the studies described in this review have been obtained from animals lacking this enzyme (e.g., rodents). These studies primarily supplement with carnosine; therefore, elevated plasma carnosine may be partly responsible for any beneficial effects shown. Rodents have low endogenous concentrations of l-histidine, potentially explaining why these studies chose to supplement with carnosine as there may be inadequate levels of l-histidine available for synthesis with β-alanine. Second, the anatomical, metabolic, and functional differences between non-primate mammals and humans must be considered. Despite these limitations, animal models are critical to study myocardial tissue and function due to the extreme difficulty in obtaining human myocardial tissue and the obvious ethical implications of inducing myocardial dysfunction in humans.

### Summary

The literature suggests that carnosine fulfills different roles within the heart, depending upon the environment (healthy or diseased). Many of the studies in the literature have exposed animal myocardial tissue to either a healthy or diseased environment. The cumulative findings from these studies indicate that carnosine improves calcium handling and, therefore, muscle contractility in a healthy model and improves oxidative stress, myocardial injury markers, and morphological and histological parameters in a diseased model. Future research should include supplementation protocols in both healthy and diseased myocardial models to determine the specific roles of carnosine in both of these conditions.

β-alanine supplementation is commonly used to increase myocardial carnosine concentrations; however, it could also be implemented to potentially decrease myocardial taurine concentration. A concentration of >3% β-alanine supplementation has been suggested to be required to reduce myocardial taurine concentration (116). There is a lack of consensus in the literature regarding the effect of taurine depletion on myocardial function and health, with rodent studies reporting beneficial (47), harmful (48, 49, 51), and even no effects (98). The decrease in myocardial taurine concentration is likely dependent upon a combination of the dose and the length of time of β-alanine supplementation. Lake and De Marte (67) showed no difference in myocardial taurine concentrations between control rats and rats supplemented with β-alanine for 6 wk, and Allo et al. (47) showed myocardial taurine concentrations reached a steady state after 2 wk of β-alanine supplementation. The fact that no changes in human skeletal muscle taurine content have been shown, despite downregulation of TauT after 24 wk of β-alanine supplementation, suggests that skeletal muscle has some way of maintaining taurine homeostasis during periods of elevated β-alanine availability (39). Further investigations into the potential complications of β-alanine supplementation on human myocardial taurine concentrations are required, although limited data suggest that the recommended doses of β-alanine are safe for human consumption (75).

## Future Perspectives

The need for long-term randomized controlled clinical trials to confirm, or not, the beneficial effects of carnosine on myocardial function and health has been highlighted in recent literature (117, 118). Lombardi et al. (119) are the only group to have conducted a clinical human trial on carnosine supplementation and heart function. They investigated 6 mo of l-carnosine supplementation in patients with stable chronic heart failure and impaired left ventricular function. The participants were supplemented with 500 mg/d l-carnosine in an open-label approach. The control group received their regular, routine treatment. l-carnosine improved quality of life and exercise capacity, but no improvements were shown in myocardial function measurements. The authors suggested that the buffering effect of l-carnosine improved exercise capacity, although this was not directly measured. The potential underlying mechanisms that might support a role for carnosine in the myocardium were not measured. The supplementation protocol contains several limitations. The lack of blinding and placebo supplement in the control group means that the study is at a high risk of bias. Participants were supplemented with intact l-carnosine, which has low bioavailability and stability in circulation due to the presence of CN1 and, in turn, will limit the uptake of carnosine into myocardial tissue. In addition, it is uncertain, and highly unlikely, that carnosine can be directly taken up intact by the myocardium. Intact carnosine is likely to be hydrolyzed into β-alanine and l-histidine, with these constituent amino acids being taken up into the tissue and re-synthesized to carnosine. An appropriate supplementation dose would be required to ensure adequate metabolite is available to the myocardium. Harris et al. (6) showed that a high dose of l-carnosine (13 g/d) is required to be able to obtain a similar increase in skeletal muscle carnosine content to that shown with 6.4 g/d of β-alanine. This suggests that the level of substrate available to the myocardium from a dose of 500 mg of l-carnosine may not be sufficient to have an overall effect on myocardial function. The recommended daily amount of β-alanine is 3.2 to 6.4 g/d for improvements in exercise capacity and performance (4, 75, 116), although potential therapeutic effects could be realized with lower doses.

Given these limitations and the insufficient evidence in human populations, higher quality clinical trials are warranted (94). Future clinical studies should incorporate a placebo-controlled, double-blinded, randomized controlled protocol. Although the impact of an increased dietary intake of β-alanine and/or carnosine through the consumption of dietary rich sources on myocardial tissue carnosine stores and health would be interesting to examine, it could also mean an increased meat intake, which might have other health implications for some. As such, it might be that a supplementation approach is preferred—with supplementation doses typically being 3.2–6.4 g/d of β-alanine in a sustained-release formula across current studies, although many of these studies were targeting improved skeletal muscle performance rather than wider health implications. From a therapeutic perspective, it is possible that lower doses (1.6 or 2.4 g/d) would be equally effective, particularly if supplementation was being considered over a longer duration than used during most studies, but all of this remains to be determined. A sustained-release formula may increase supplementation adherence as participants will be less likely to experience the common side effect of paraesthesia (i.e., tingling), although this might also be achieved at lower doses of a non-sustained-release formula. Implementing a supplementation intervention in a heart failure population is exceptionally challenging. There are many factors to consider, including feasibility, patient access, and side effects. Therefore, future human studies may evaluate the effectiveness of carnosine on myocardial function in clinical populations with an associated cardiovascular disease risk comorbidity (e.g., a prediabetic or aging population) before conducting interventions in higher-risk individuals.

## Conclusions

Carnosine has the potential to improve healthy and diseased myocardial function, although there is still a significant gap in our understanding and application of carnosine within the cardiovascular system. More research is needed to identify the exact roles carnosine plays in the myocardial tissue in healthy and diseased models. It is possible that carnosine functions differently depending upon the tissue's environment, although further research is needed to clarify the distinct differences between these environments in cell, animal, and human experiments. The translation of findings into the human population is limited, primarily due to rodent models lacking the enzyme that degrades carnosine in humans (CN1). Practical recommendations for β-alanine supplementation cannot yet be given concerning myocardial function and health.

## Notes

The authors report no specific funding received for this work.

Author disclosures: JVC is the recipient of a PhD studentship funded by Natural Alternatives International (NAI). DT is the Director of Scientific Affairs at NAI. CS is the recipient of funding to support a PhD program of work from NAI for JVC (as described above). CS and GGA have received β-alanine supplements free of charge from NAI for use in experimental investigations. NAI has also supported open-access page charges for some of CS and GGA manuscripts and CS has also received payment from NAI for the production of material to support a blog post. MDT received a British Council grant award to support a PhD studentship focused on research into carnosine (grant number s209524711). All other authors report no conflicts of interest.

Abbreviations used: AGE, advanced-glycation end product; AGXT2, alanine-glyoxylate aminotransferase 2; ALE, advanced lipid end product; CARNS1, carnosine synthase; DOX, doxorubicin; EC, excitation-contraction; ECG, electrocardiogram; GABA-T, 4-aminobutyrate-2-oxoglutarate transaminase; HCD, histidine-containing dipeptide; HNE, 4-hydroxy-2-nonenal; MSA, malonate semi-aldehyde; PAT1, proton-coupled amino acid transporter; ROS, reactive oxygen species; RyR, ryanodine receptor; TauT, taurine transporter.
